# User-Centered Diabetes Self-Management App (DiabAid Nexus) in Sub-Saharan Africa: Development and Usability Study

**DOI:** 10.2196/87110

**Published:** 2026-06-02

**Authors:** Genet Tadese Aboye, Ermias Habte Gebremichael, Gizeaddis Lamesgin Simegn, Jean-Marie Aerts

**Affiliations:** 1 Department of Biosystems, Division of Animal and Human Health Engineering, M3-BIORES (Measure, Model & Manage Bioreponses), KU Leuven, Kasteelpark Arenberg 30, Leuven, 3000, Belgium, +251945046309; 2 School of Biomedical Engineering, Jimma University, Jimma, Ethiopia; 3 Department of Internal Medicine, Jimma University, Jimma, Ethiopia; 4 The Russel H. Morgan Department of Radiology and Radiological Science, Johns Hopkins University, Baltimore, MD, United States

**Keywords:** diabetes, low-resource settings, mHealth, self-management, user-centered design, mobile phone

## Abstract

**Background:**

Diabetes is a significant global public health concern, with disproportionately high prevalence and poor access to care in low-resource settings, notably in Sub-Saharan Africa.

**Objective:**

By harnessing the ubiquity of mobile phones, we aim to develop and test a user-centered mobile health (mHealth) app tailored to address the specific needs and challenges of diabetes self-management in Sub-Saharan Africa.

**Methods:**

A user-centered design approach was used to develop a bilingual mHealth platform for diabetes management. The app was made using Flutter 3.16.2 (Google) and Dart 3.2.2. (Google) and was tested on various Android emulators and smartphones before publishing. A usability study with 13 participants was carried out to assess the app’s real-life impact, quality, and user experience using the user version of the Mobile App Rating Scale (uMARS). A brief semistructured interview was conducted to further evaluate the app’s usability and user experience.

**Results:**

The mHealth platform called “*DiabAid Nexus*” (GTA) has been developed and tested with 13 people living with diabetes in Ethiopia. The findings show that the app was perceived as simple, easy to use, and relevant to users’ needs. uMARS scores out of 5 showed high ratings for functionality (mean 4.33, SD 0.63), information quality (mean 4.20, SD 0.68), perceived impact (mean 4.20, SD 0.84), and subjective quality (mean 4.10, SD 0.73), indicating that users deemed the app useful, dependable, and valuable for diabetic self-management. The aesthetics subscale likewise obtained a positive score (mean 4.05, SD 0.94), while engagement earned a slightly lower score (mean 3.78, SD 0.95), highlighting the possibility of further boosting interactivity and user motivation. The overall uMARS score of 4.10 suggests a favorable user assessment of the app’s quality and efficacy. Interview responses underlined appreciation for localized content and intuitive design, with users indicating happiness and a readiness to use the app as a tool to support their diabetes self-management journey.

**Conclusions:**

The findings suggest that the app is a user-friendly and well-received tool with a high potential for promoting diabetes self-management in low-resource settings and across varied contexts.

## Introduction

The global burden of noncommunicable diseases continues to rise, with diabetes emerging as one of the most challenging chronic conditions to manage, particularly in low-resource settings (LRSs) [1,2]. Diabetes mellitus is a clinical condition comprising a heterogeneous group of metabolic diseases that are characterized by chronic hyperglycemia and disturbances in carbohydrate, fat, and protein metabolism secondary to defects in insulin secretion, insulin action, or both [3]. Without effective management, it poses significant challenges and can lead to severe complications [4,5].

Over the last 3 decades, the worldwide diabetes burden has increased rapidly. In 2021, approximately 537 million adults had diabetes, with this number expected to rise to 643 million by 2030 and 783 million by 2045 [6]. Notably, this increase has been more pronounced in low- and middle-income countries than in high-income nations, highlighting the growing inequities in illness distribution and management. Diabetes prevalence among individuals aged 18 years and older in the World Health Organization’s African Region has nearly doubled in just over 30 years, from 6.4% in 1990 to 10.5% in 2022 [2,7]. Despite the rapid increase, access to care remains limited. As of 2022, only 26.1% of adults aged 30 years and older diagnosed with diabetes in the African Region received treatment, leaving almost 34 million people without proper medical care [7,8]. Diabetes is increasingly managed across decades, and older adults may face added self-management challenges due to multimorbidity, vision changes, reduced manual dexterity, and heightened vulnerability to severe hypoglycemia. All of these can complicate diabetes management, reinforcing the value of simple, accessible mobile health (mHealth) support across the patients’ lifespan [9]. Managing diabetes is particularly difficult in resource-constrained settings, where the health care system is overburdened, patients often face limited access to health services, insufficient diabetes education, and a lack of tools to support effective self-management [10,11].

mHealth platforms are gaining traction for their ability to improve equity in health care delivery across diverse contexts, including low-resource environments. They show considerable promise in managing chronic diseases, such as diabetes [12-15]. A key advantage of mHealth is its capacity to extend health care access to geographically isolated and underserved communities, thereby reducing service inequities. In diabetes care, mHealth interventions have been shown to improve glucose control, medication adherence, and self-care practices [16]. Moreover, a growing body of evidence highlights their potential to enable continuous patient interaction, real-time feedback, and individualized support [17,18]. However, their effectiveness depends heavily on usability, cultural relevance, and alignment with user expectations and digital literacy levels [19]. For mHealth solutions to be embraced and maintained in regular use, they must be perceived as functional and accessible by their intended audience [20,21].

Digital health literacy is another critical determinant of success. Many users in LRSs lack experience with smartphones, reliable internet or data access, or confidence in navigating health apps. A scoping review of mHealth and telemedicine systems for gestational diabetes reported that only about 12% (2/17) of reviewed apps incorporated culturally appropriate design features, and just 25% (3/12) adequately addressed digital health literacy [22]. Such gaps hinder adoption and long-term use. User perceptions also play a central role. Factors such as usability, design aesthetics, interactivity, and motivational features significantly influence engagement [23]. For instance, a qualitative study of adults with type 2 diabetes in Sweden [24] found that participants valued interactive features, varied functions, and user-friendly layouts as essential for long-term use and behavior change. They emphasized the importance of holistic, goal-oriented content and integration of social support. Clinical trials further reinforce the benefits of mHealth. In India, a randomized controlled trial involving 100 patients with type 2 diabetes showed that combining routine care with weekly phone calls and messages significantly improved hemoglobin A1c (HbA_1c_) levels, medication adherence, diet, and physical activity compared with routine care alone [25]. Similarly, a meta-analysis focusing on older adults found that mHealth apps led to a significant reduction in HbA_1c_, suggesting effectiveness even in populations often assumed to have lower digital literacy [26].

Importantly, mHealth interventions are not universally beneficial. Their impact often varies according to socioeconomic and sociocultural factors that shape access and outcomes. A recent systematic review noted that few trials of mHealth apps for diabetes and hypertension accounted for differences across sociodemographic groups, such as income, education, gender, or rural versus urban residence [21]. Consequently, mHealth can either help to reduce or inadvertently reinforce health inequities, depending on how interventions are designed and implemented.

These challenges and opportunities highlight the urgent need for innovative, accessible, and scalable solutions to support diabetes care and self-management, particularly in resource-limited settings. At the same time, they point to the importance of empowering individuals to take an active role in managing their health.

Leveraging the widespread availability of mobile phones, this study seeks to design and evaluate a user-centered mHealth platform tailored to the unique challenges of diabetes self-management in Sub-Saharan Africa. The intervention was developed using a user-centered approach guided by the mHealth Agile Development and Evaluation Lifecycle. This paper reports on the design, development, and early evaluation of the platform, emphasizing simplicity, cultural contextualization, and iterative user feedback. By integrating evidence-based design principles with direct input from end users, the study aims to advance the development of effective, context-appropriate digital health interventions.

## Methods

### Overview

A user-centered mobile app development approach was used, guided by the mHealth Agile Development and Evaluation Lifecycle framework [27]. This framework comprises a project identification stage, followed by four sequential phases of clinical evaluation (1) phase 1: user experience design, development, and alpha testing; (2) phase 2: beta testing; (3) phase 3: clinical trial evaluation; and (4) phase 4: postmarket surveillance. In the context of this study, implementation was carried out up to phase 2. This study is reported in accordance with the mHealth evidence reporting and assessment (mERA) checklist.

### Phase 1: User-Centered App Design and Development

#### Overview

We used the insights from our previous works [28,29], which highlighted the challenges and unmet needs of individuals living with diabetes, as well as the barriers and facilitators to using mHealth approaches in the Sub-Saharan Africa context. These insights were translated into design features, and we subsequently developed *DiabAid Nexus*, an Android-based smartphone app for diabetes self-management.

#### Case: A Typical Routine of Diabetes Diagnosis, Management, and Coping in the Life of a Person Living With Diabetes in LRSs

**Figure 1.**
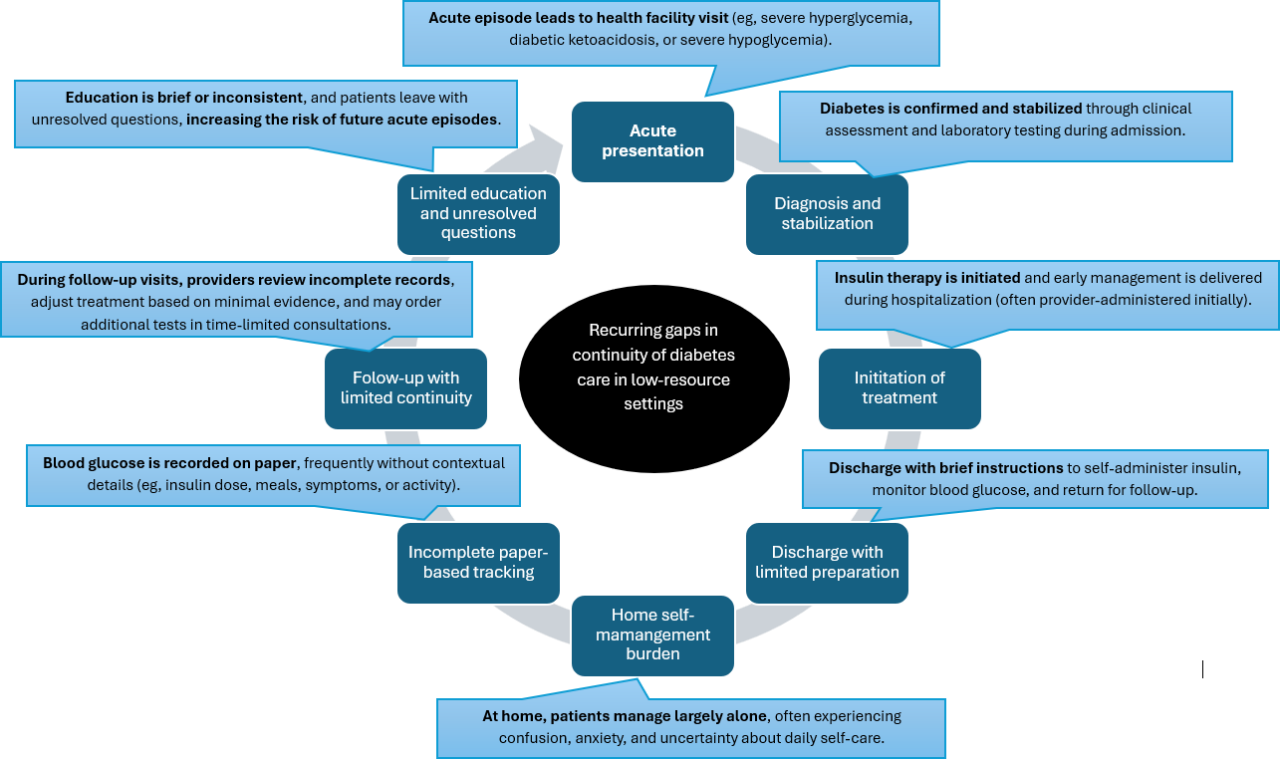
A typical routine of diabetes diagnosis, management, and coping in the life of a person living with diabetes in low-resource settings.

A typical routine of diabetes diagnosis, management, and coping in the life of a person living with diabetes in LRSs, identified through observation and patient interview, presented in Figure 1, illustrates systemic challenges in diabetes care in Ethiopia, including delayed diagnosis, limited patient education, reliance on incomplete paper records, rushed consultations, and patients’ ongoing confusion and anxiety about self-management. These gaps contribute to poor continuity of care, inadequate glycemic control, and heightened risk of long-term complications.

#### Proposed App Features

**Figure 2.**
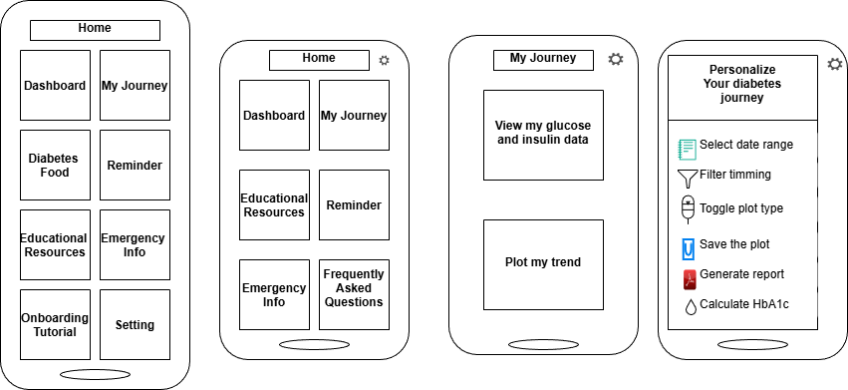
Sample low-fidelity wireframes that illustrate the iterative design process of the DiabAid Nexus app.

**Figure 3.**
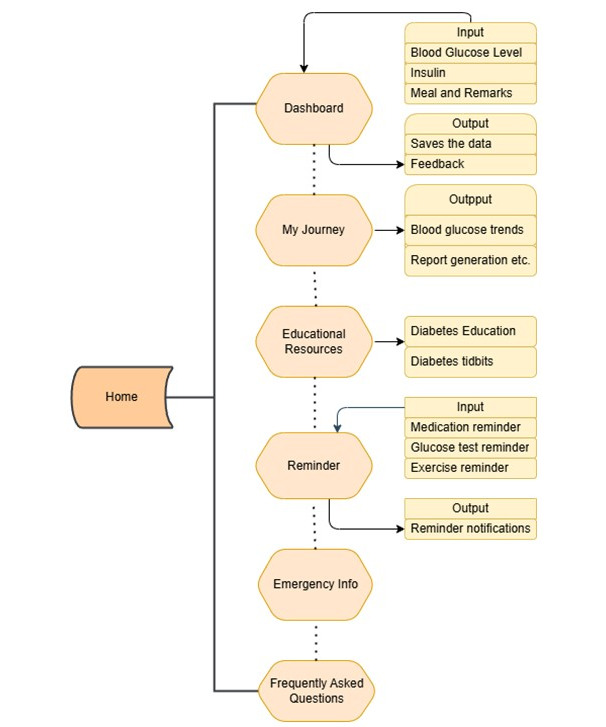
Flow diagram representing the *DiabAid Nexus* app key features.

*DiabAid Nexus* was specifically designed to address these challenges by providing patients with accessible, structured, and user-centered tools for diabetes self-management. The low-fidelity wireframes illustrated in Figure 2 and the flow diagram presented in Figure 3 illustrate the main features, subfeatures, and wireframing of the app, which were derived from the case described above. Building on this design, a comprehensive diabetes self-management mHealth platform available both in English and Amharic, *DiabAid Nexus*, was developed.

The main features of the app consist of Dashboard, My Journey, Educational Resources, Reminders, Emergency Info, and Frequently Asked Questions.

First, the “Dashboard” replaces the traditional paper-based glucose logbooks, enabling patients to electronically record glucose values, insulin dosages, meal intake, and contextual remarks. This not only ensures more complete and accurate data but also addresses the issue of undocumented insulin adjustments, which are common among syringe users. By centralizing this information, the app reduces patient confusion and provides health care providers with a more reliable basis for clinical decision-making.

Second, the “My Journey” feature empowers patients to visualize and personalize their diabetes data. Unlike paper records that provide only isolated glucose values, this tool generates blood glucose trend analyses, highlighting periods of out-of-range glycemic values, and allowing patients to associate these events with lifestyle factors, such as meals or exercise. The blood glucose trend graph illustrates date on its x-axis and blood glucose value (in mg/dL or mmol/dL) on its y-axis. The blood glucose trend plot can be customized to an aggregated plot or a detailed plot. The user can also apply filters to select specific blood glucose reading timings, such as fasting, random, after lunch, and so on. Moreover, additional options also exist for estimating the HbA_1c_. If there are no (or not enough) inputs provided, it will display “No data to show.” The ability to generate PDF summaries and HbA_1c_ estimates further supports continuity of care by enabling patients to share structured data with their health care professionals, thus reducing the limitations of rushed consultations and incomplete follow-up information.

Third, the “Educational Resources” directly address the widespread gaps in patient education observed in case 1. By providing clinically validated, comprehensive, and accessible content in both English and Amharic, the app ensures that patients gain a deeper understanding of diabetes, its treatment, and strategies for risk reduction. The contents were initially drafted based on the national and international standard guidelines for Diabetes Self-Management Education [30,31]. Importantly, the content helps alleviate fear and uncertainty by clearly explaining complications and offering guidance on prevention, thereby counteracting the worry, stigma, and misinformation that often undermine self-management.

Fourth, the “Reminder” function supports adherence by prompting patients to test glucose, administer insulin, and exercise at the appropriate times. This mitigates the common problem of missed or delayed medication, which contributes to poor glycemic control. Reminders are delivered via scheduled push notifications. Alerts follow the device’s default notification settings, including sound. If the phone is set to silent mode, the reminder will appear as a visual notification without sound, in accordance with the device’s operating system behavior.

Fifth, the “Emergency Info” feature allows patients to store vital medical and contact information for use during acute episodes. This addresses the high-risk scenario of presenting to health facilities in critical condition (eg, diabetic ketoacidosis or severe hypoglycemia) by ensuring that health care providers and caregivers have immediate access to relevant information.

Finally, the “Frequently Asked Questions” section complements formal education by answering common questions in a patient-friendly format, helping to resolve confusion that typically persists after brief and incomplete consultations.

Overall, by digitizing record-keeping, enhancing patient education, improving adherence, and facilitating communication between patients and health care professionals, *DiabAid Nexus* directly targets the weak points identified in the observed cycle of diabetes management. The app therefore holds potential not only to reduce patient anxiety and improve self-efficacy but also has the potential to support continuity of self-management between clinical visits.

After the initial wireframing stage, the preliminary app design was presented to 3 individuals living with diabetes who served as patient subject matter informants to obtain early feedback and guidance. Feedback was collected through individual, structured consultation sessions (one-on-one discussions guided by the wireframes and core user flows), focusing on usability, accessibility, and perceived relevance. Suggestions from these sessions were incorporated iteratively to refine the design before proceeding to system development.

#### System Development

The implementation made use of Flutter (version 3.16.2; Google), which serves as a cross-platform user interface software development toolkit, in combination with Dart (version 3.2.2; Google) [32,33], the primary programming language used to build the app’s functionality. Flutter provided the framework for rendering consistent and responsive user interface components across multiple platforms, while Dart enabled the creation of efficient, object-oriented program logic. The development activities were carried out within Android Studio (Google), which functioned as the integrated development environment, offering debugging, emulation, and code management features.

The app supports English and Amharic (widely spoken in Ethiopia). Users select their preferred language in the settings menu, and the choice is saved locally via shared_preferences so it persists across sessions. All user-facing content (eg, titles, labels, snackbars, and educational text) updates automatically when the language changes. The app was developed first in English, then all visible strings were translated into Amharic using Google Translate, followed by manual review. Language switching was implemented using Flutter localization keys with separate .arb resource files for each language, which Flutter compiles into the corresponding generated localization files.

Upon the completion of the iterative development and preliminary testing cycles, the app was prepared for controlled distribution. To facilitate further evaluation under real-world conditions, the app was deployed to the Google Play Store in internal testing mode, thereby allowing designated testers to access and assess its performance before wider public release.

#### Security and Privacy Measures

The app uses Firebase Authentication (Google) to manage user registration and login [34]. Only a username is collected during registration, thereby minimizing the amount of personal data stored. Login activity, such as the number of logins within a given period of time, is recorded to support usage monitoring and system evaluation. All authentication processes are securely handled by Firebase, which uses encrypted communication (HTTPS) and industry-standard hashing for credential management. By limiting the scope of collected data and relying on Firebase’s built-in security mechanisms, the app ensures user privacy while maintaining reliable access control.

#### Cost and Resource Requirements

DiabAid Nexus was developed in-house by the research team using open-source tools (Flutter and Dart), minimizing software licensing costs. The app was deployed via Google Play internal testing and uses Firebase Authentication. During the study, usage remained within the free-tier limits with no added hosting costs. The app is free for users with no subscription fees and functions largely offline after installation, requiring internet mainly for download, authentication, and updates, which helps limit data costs. No formal economic evaluation was conducted, but future scale-up may require resources for hosting, maintenance, content updates, and user support.

### Phase 2: Usability Testing

#### User Version of the Mobile Application Rating Scale

A total of 13 eligible individuals (Table 1) participated in the usability evaluation of the mobile app. The participants were people living with diabetes, residing in Jimma and Addis Ababa, Ethiopia. These cities are among those in Ethiopia with broad access to the internet and electricity coverage [35]. Participants were first contacted through a contact person at the Ethiopian Diabetes Association (Addis Ababa) and a diabetes clinic at Jimma University Medical Center. Eligible individuals were informed about the study and invited to participate. Purposive sampling was used to include adults living with diabetes who owned an Android (Google) smartphone and were willing to engage with the app during the study period. Previous exposure to diabetes-related apps was not used as an eligibility criterion. The participants evaluated the usability of the mobile app in terms of engagement, functionality, aesthetics, information, and the app’s subjective quality. Participants who consented to take part in the study had their Google account registered, and a link was sent to them to download the app on their mobile phones. Following a brief onboarding session that provided guidance on the app’s features and functionality, they were instructed to engage with the app over a minimum period of 8 weeks (from March to May 2025). At the end of this period, participants were invited to complete the user version of the Mobile Application Rating Scale (uMARS) and take part in a brief semistructured interview to further explore their experiences and perspectives. The uMARS is a validated tool with a 26-item measure that includes subscales to assess engagement, functionality, aesthetics, and information quality of the app [36].

**Table 1.** Demographic characteristics of participants (N=13) involved in the 8-week usability evaluation of the DiabAid Nexus app conducted in Addis Ababa and Jimma, Ethiopia (March-May 2025).

Characteristics	Participants (N=13), n (%)
Sex	
Male	10 (76.9)
Female	3 (23.1)
Age (y)	
18‐24	7 (53.8)
25‐34	5 (38.5)
35‐44	1 (7.7)
Location	
Addis Ababa	7 (53.8)
Jimma	6 (46.2)
Level of education	
Secondary or high school	1 (7.7)
Technical or vocational training	1 (7.7)
University or college degree	10 (76.9)
Postgraduate degree	1 (7.7)
Employment status	
Employed (full-time or part-time)	6 (46.2)
Self-employed	1 (7.7)
Unemployed	1 (7.7)
Student	4 (30.8)
Other	1 (7.7)

#### Interview

After testing the app and rating it with the uMARS scale, participants were asked questions to qualitatively learn their overall impression, likes and dislikes, feature usage, impact on self-management, and cultural and contextual fit.

### Data Analysis

After a systematic design and iterative enhancement process, the final iteration of the mobile app was launched on the Google Play Store for internal evaluation. Following the conclusion of the usability evaluation session, participants used Google Forms to submit their answers to the uMARS. For preprocessing, the gathered data were exported to Microsoft Excel. Data cleansing was done by standardizing variable names, transforming categorical replies into numeric codes using the uMARS Likert scale, and guaranteeing completeness and consistency. No incomplete submissions were included. Each participant’s responses to the corresponding items were averaged to determine their subscale scores, which included engagement, functionality, aesthetics, information quality, subjective quality, and perceived impact. The core subscale scores were also averaged to determine an overall usability score. Only descriptive statistics were performed due to the exploratory nature and small sample size. Inductive thematic analysis and coding of the semistructured interview were also used to get deeper insights into user feedback during the beta testing period. In total, 2 researchers (GTA and GLS) were involved in the coding collaboratively, and discrepancies were resolved through discussion. A third researcher (JMA) reviewed the finalized themes.

### Ethical Considerations

The study protocol was approved by the KU Leuven Research Ethics Committee (G-2024‐8194) and the Institutional Review Board of Jimma University Institute of Health (JUIH/IRB/311/23). All participants provided written informed consent before participation. To protect participant privacy and confidentiality, all study data were deidentified before analysis, kept in a secure network storage, and handled using appropriate safeguards to prevent reidentification. Participants received compensation of 25 Euros (US $29.25) for their participation.

## Results

### Design Implementation

**Figure 4.**
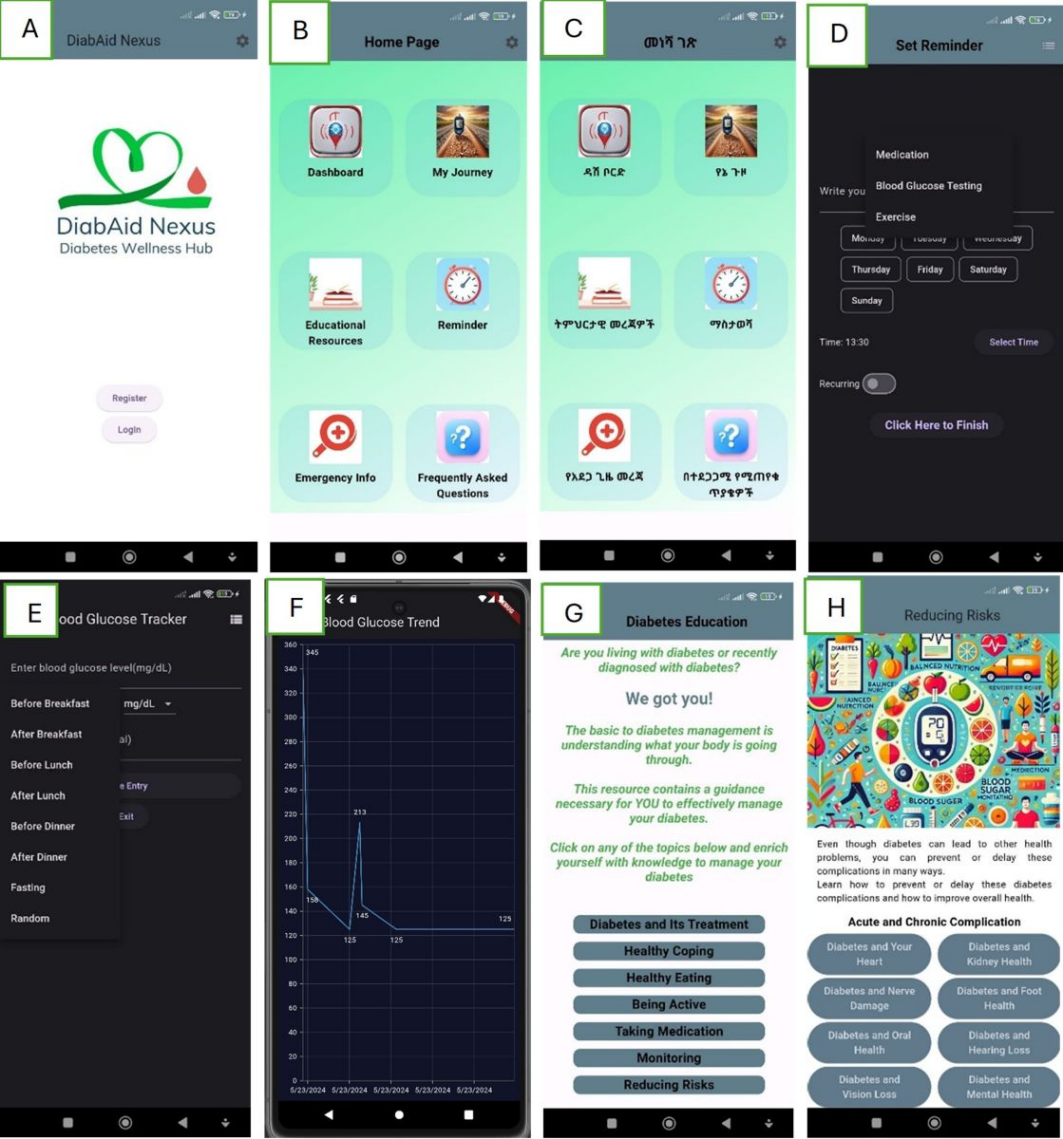
Sample screenshots for the DiabAid Nexus app. (A) Login and Registration page, (B) Home page (English), (C) Home page (Amharic), (D) Reminder, (E) Blood glucose logging, (F) Blood glucose trend plot (G) Diabetes Education main page, and (H) Education for reducing risks of acute and chronic complications.

These design features were presented and discussed with various people living with diabetes. After a consensus was reached, the app was developed using Flutter and Dart in Android Studio. The app has been implemented and tested on Android (version 14) devices as well as multiple emulators in preparation for the formal usability testing phase. The design and layout of the app’s core functionality are illustrated in Figure 4.

### Usability Study or Beta Testing

#### Study Sample

Table 1 presents the overall characteristics of the participants who participated in the real-life usability test of the app. Recruitment was conducted with the assistance of contact persons from Jimma University Medical Center (Jimma) and the Ethiopian Diabetes Association (Addis Ababa).

**Figure 5.**
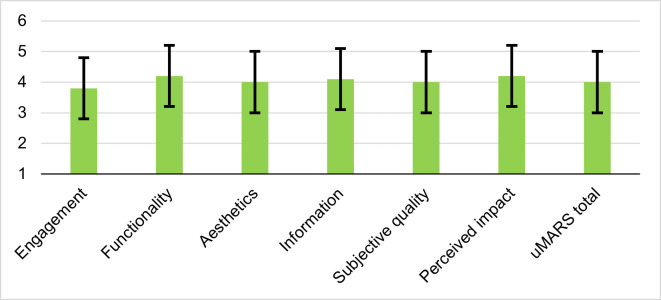
.Mean uMARS subscale scores for the DiabAid Nexus app during an 8-week real-world usability assessment in Ethiopia. Scores are based on evaluations from 13 participants and are reported on a 1–5 scale. Error bars indicate SDs. uMARS: user version of the Mobile App Rating Scale.

#### uMARS

Functionality had the highest mean score (mean 4.33/5, SD 0.63), with a minimum of 3.25/5 and a maximum of 5.00, indicating excellent performance in terms of ease of use, navigation, and general technological reliability. Information quality and perceived impact both scored high (mean 4.20/5, SD 0.68; mean 4.20/5, SD 0.84, respectively), indicating that the app was perceived as informative and potentially beneficial in influencing user behavior. Aesthetics (mean 4.05/5, SD 0.94) and subjective app quality (mean 4.10/5, SD 0.73) indicate positive feedback in terms of visual appeal and overall user satisfaction. The engagement subscale had the lowest mean score (mean 3.78/5, SD 0.95), with scores ranging from 2.20/5 to 5.00, indicating more variation in users’ experiences with the app’s interactivity and motivational aspects, and highlighting potential for growth in terms of interactivity and motivation to use the app on a regular basis. The overall uMARS total score was 4.10/5 (SD 0.76), indicating that users found the app to be of high quality and effective in supporting its intended purposes. The overall uMARS result is presented in Figure 5.

#### Interview Results

The interview results, presented in Table 2, highlight participants’ generally positive perceptions of the app, including its usefulness, ease of navigation, self-management support, and suggestions for further improvement.

**Table 2.** Selective illustrative quotations about the overall impressions, likes and dislikes, feature usage, and cultural fit of the app as perceived by the participants.

Theme	Illustrative quotation
Overall impressions of the app	“It is a very good and useful app.” [P01^a^] “Initially, I did not expect the app to include such a wide range of features, especially considering its localization. I was not anticipating a highly developed platform. However, what I observed was quite impressive. The app is very well-designed.” [P02]
Likes and dislikes	“Overall the app does not have confusing part. But I think it will be better if the graphic designs are improved more.” [P02] “The app is quite simple and easy to navigate, even for people who are not very familiar with technology.” [P07]
Mostly used features	“I personally found the reminder very helpful and I used it a lot. For people with a tight schedule, we tend to forget a lot. I tend to forget my insulin shots. So the reminder helped a lot.” [P02] “I used the dashboard to register my glucose levels. It will help me significantly during my doctor visits. Previously, I recorded my measurements on paper, but now I use the app to electronically log my readings and visualize how my blood sugar has been over time.” [P013]
Impact on self-management	“I educated me a lot. It encouraged me do physical exercise daily and also share my condition with people I love.” [P01] “The fact that I can log detailed events such as the meals and remarks, in addition to my blood glucose levels, is very helpful.” [P08]
Recommendations	“I would love the app to incorporate detail food guide/meal guide.” [P06] “I suggest the app periodically provide new information/findings about diabetes.” [P011]

a P01: patient 1.

## Discussion

### Principal Findings

In this paper, we presented a user-centered design and development of *DiabAid Nexus*, a mHealth app developed to support diabetes self-management. The app is specifically designed to address the challenges and barriers faced by individuals living with diabetes, particularly in LRSs and environments with limited access to continuous monitoring, such as those commonly found in Sub-Saharan Africa.

The typical routine case of diabetes diagnosis, management, and coping in the life of a person living with diabetes in LRSs is highlighted in Figure 1. It shows persistent weaknesses in Ethiopia’s diabetes care delivery, including limited patient education, incomplete paper-based monitoring, rushed consultations, and inadequate continuity of care. Despite national efforts to strengthen services, individuals are often left without essential resources for effective care. These challenges mirror those reported in other resource-limited settings, where patients often present after acute complications and leave health facilities without a sufficient understanding of diabetes as a lifelong condition [1,37,38]. Such gaps undermine self-management, increase the risk of poor glycemic control, and contribute to complications and psychosocial distress.

*DiabAid Nexus* was developed in direct response to these shortcomings, offering patient-centered tools that strengthen self-management outside the clinic setting. By replacing paper-based glucose logs with structured digital entries and allowing patients to track insulin use, meals, and contextual factors, the app provides individuals with a clearer picture of their own health patterns. The ability to view trends and generate summaries supports patient reflection and enhances their capacity to engage more meaningfully during follow-up visits, even when consultations are brief. Comprehensive, clinically validated educational resources embedded in the app further address the shortage of structured diabetes education, equipping patients with practical knowledge on treatment, lifestyle modification, and complication prevention. In addition, reminders and emergency information features support adherence and safety in day-to-day life.

A notable strength of *DiabAid Nexus* lies in its bilingual design. By providing content and functionality in both English and Amharic, the app reduces language barriers that frequently prevent patients from fully understanding medical advice or self-management guidelines. This feature expands accessibility beyond urban, educated populations to include a broader demographic, thereby promoting equity in digital health interventions. In a context where low health literacy and language diversity limit the effectiveness of traditional education, bilingual delivery enhances comprehension, empowers patients to take ownership of their condition, and reduces reliance on overburdened clinical encounters [39].

Taken together, these features position *DiabAid Nexus* as a contextually tailored, patient-empowering mHealth tool. Rather than replacing healthcare providers, it complements the limited support available within the health system by strengthening patients’ ability to manage their condition and fostering self-efficacy.

While numerous diabetes management apps exist globally (eg, *Blood Sugar Diary* (MedM Inc) [40], *mySugr - Diabetes Tracker Log* (mySugr GmbH) [41], *WeCare Diabetes* (MEGA LIFESCIENCES) [42]), many are optimized for high-resource contexts and often emphasize a single function (eg, logging or education) [40,42] or assume access to advanced technologies, such as insulin or continuous glucose monitoring systems [41,43]. *DiabAid Nexus* is distinct in both design rationale and scope. To our knowledge, it is the first mHealth app developed specifically in response to the authentic, unmet needs of individuals living with diabetes in Ethiopia, as identified through direct user engagement and contextual research. In terms of contextual adaptation, the app provides bilingual content (English and Amharic), thereby enhancing accessibility for users with limited proficiency in global languages, and delivers clinically validated education in a format intended to reduce fear, misinformation, and stigma that can undermine self-management. In terms of functionality, DiabAid Nexus goes beyond replacing paper logbooks; its dashboard supports structured capture of glucose values alongside insulin doses, meal intake, and contextual remarks. This helps address common gaps, such as undocumented insulin adjustment among syringe users. The My Journey module further differentiates the app by enabling customizable trend visualization (fasting vs postprandial filters), generating structured summaries (including PDF export and HbA_1c_ estimation) to support continuity of care during brief consultations. Finally, unlike many apps that focus on a single component, *DiabAid Nexus* integrates tracking, tailored education, reminders, emergency information, and FAQs into 1 user-centered platform designed for resource-constrained settings. A feature-level comparison with diabetes mHealth apps is provided in Table 3 to highlight differences in functionality, contextual adaptation, and intended user population.

**Table 3.** Comparison of DiabAid Nexus with typical commercial diabetes apps.

Feature	DiabAid Nexus	Typical commercial diabetes apps
Electronic glucose logging	✔	✔
Insulin dose documentation	✔(Explicitly addresses undocumented adjustments)	Partial
Trend visualization with filters	✔(Customizable and fasting, random, or postprandial filters)	✔
HbA1c^a^ estimation	✔	✔
PDF export for clinical visits	✔	Sometimes
Offline or low-data functionality	✔(Designed for low-resource settings)	Often requires a stable internet
Local language support	✔(English+Amharic)	Rare
Context-specific education	✔(Culturally tailored, stigma-sensitive)	Generalized content
Community-oriented emergency info	✔	Rare

a HbA1c: hemoglobin A1c.

DiabAid Nexus also aligns with lessons from successful Sub-Saharan African mHealth initiatives. Wali et al [44] describe the community-based adaptation of Medly Uganda heart failure program, highlighting user-centered design, cultural language tailoring, and integration with trusted community health structures to bridge remote communities and clinical care. Similarly, DiabAid Nexus was developed to address context-specific barriers, such as fragmented follow-up and limited patient education, underscoring that effective mHealth in LRSs depends on contextual adaptation and health system alignment rather than technological complexity alone.

The usability evaluation of the mobile app using the uMARS shows that participants generally liked the app, with consistently high scores across key domains. The highest-rated subscale was functionality, indicating that users judged the program to be dependable, simple to use, and technologically sound. This is an important component of digital health interventions, especially in resource-constrained contexts where technological challenges might impede user engagement and long-term adoption [28,45,46]. The high functionality score indicates that the design and development process successfully prioritized user experience and performance stability.

The high ratings for information quality and perceived impact indicate that users found the app’s material to be accurate, relevant, and beneficial in shaping their knowledge or self-management habits. This is a positive result, particularly for a digital health tool aimed at individuals with limited access to individualized health education. It also aligns with the app’s primary objective of enhancing diabetic self-management, suggesting that it has the potential to positively impact patient outcomes. Studies have demonstrated the effectiveness of apps with educational content in chronic disease management [47]. Furthermore, the result supports the findings by [48] that highlighted internet health information quality plays a stronger role in impacting patients’ trust and compliance. Aesthetics and subjective quality results are supported by the research findings that gave evidence for the importance of aesthetic design in influencing good user views of online interventions, with a focus on the roles of simplicity and craftsmanship. Simplicity in design was found to have a substantial impact on users’ perceptions of ease of use, implying that streamlined, uncluttered interfaces improve accessibility and overall user experience [49]. However, there was a little greater variation in aesthetics, with some people scoring it as low as 2.00. This shows that, while the visual design is generally accepted, future revisions of the app may benefit from design changes to accommodate varied user tastes. The comparatively lower engagement score may reflect the app’s current focus on functionality and education rather than interactive or motivational features. Research in digital behavior change suggests that gamification, social interaction, and personalization may enhance long-term engagement [50]. Engagement is a significant predictor of adherence and long-term use in digital health applications. This can be achieved by integrating psychological theory while developing digital health tools [23]. As a result, future development should focus on improving this aspect, possibly by incorporating features like gamification or peer assistance functionalities to boost user engagement and retention. During the interview, no explicit negative or critical reactions were reported by participants.

In summary, the result indicates that the app is a well-designed and functional tool with the potential to assist users in self-management. Evidence from randomized controlled trials of other diabetes mHealth apps has demonstrated improvements in glycemic control [18]. While our study did not assess clinical outcomes, these findings suggest that further evaluation of DiabAid Nexus in a clinical trial setting would be warranted. Nonetheless, targeted enhancements, particularly those aimed at increasing engagement, could boost its effectiveness and user retention. These findings lay a solid platform for expanding the app and undertaking more research to determine its influence on clinical outcomes and behavioral change in real-world situations.

Despite its potential, *DiabAid Nexus* also faces several limitations that need to be considered when interpreting these findings and planning future implementation. First, the app’s effectiveness depends on long-term user engagement, motivation, and digital literacy. Participants in this study were relatively well-educated, which may limit generalizability to populations with lower (digital) literacy or limited experience with smartphones. In settings where digital literacy is lower, community-based digital health training may be required to ensure equitable access.

Furthermore, scalability may be constrained by factors such as smartphone ownership, stable electricity, and internet connectivity. While the app is designed to function largely offline after installation, initial download, authentication, and updates require connectivity. Socioeconomic disparities may also influence long-term implementation. While the bilingual design improves accessibility, it does not fully address barriers among patients with very low literacy levels or those who rely on other local languages beyond Amharic. Moreover, users are required to manually log their insulin and glucose values into the app. It is also worth documenting which language version each user evaluated. Future updates should aim to integrate features such as image recognition in order to integrate data directly from glucometers by using the camera feature of smartphones. This would simplify glucose tracking for users, reduce data entry errors by patients, and enhance interactivity and accuracy in daily monitoring. Beyond data capture, incorporating “social” and clinical communication features may enhance long-term engagement and self-management, such as secure messaging or data-sharing with health care providers for feedback, as well as optional peer or care-partner support functions. As highlighted in Azevedo et al [26], app components and long-term engagement appear to influence glycemic outcomes among older adults, suggesting that functionality beyond basic self-monitoring may enhance long-term effectiveness. Finally, as with other mHealth interventions, long-term sustainability and scalability will require strategies for integration with national digital health initiatives, and partnership with public health institutions, ongoing technical support, periodic content updates, and alignment with national health system priorities.

### Conclusions

Grounded in rigorous formative research that offers an evidence-based understanding of the contextual needs, barriers, and preferences of individuals living with diabetes in Sub-Saharan Africa, *DiabAid Nexus* mHealth app for diabetes self-management was developed based on previous formative research and user input. The alpha testing phase, conducted in collaboration with the developer team, enabled the iterative refinement of the app before formal usability testing. Adhering to a user-centered design approach throughout various stages of development further ensured that the app aligns closely with the lived experiences and expectations of people with diabetes. Finally, the use of both quantitative data from the uMARS usability survey and qualitative insights from interviews enhanced the comprehensiveness of the evaluation, suggesting that the app is both feasible and acceptable for use among diverse users living with diabetes.

## Supplementary material

10.2196/87110 Checklist 1 mERA (mHealth evidence reporting and assessment) checklist.

## Abbreviations

HbA_1c_: hemoglobin A1c
LRS: low-resource setting
mERA: mHealth evidence reporting and assessment
mHealth: mobile health
uMARS: Mobile App Rating Scale
