# Supplementary material for: User-Centered Diabetes Self-Management App (DiabAid Nexus) in Sub-Saharan Africa: Development and Usability Study
Source: J Med Internet Res. 2026 Jun 2;28:e87110. doi: 10.2196/87110 (PMC13229393; doi:10.2196/87110)
Supplement: Checklist 1 [file jmir-v28-e87110-s001.pdf]

Table 1 |mHealth evidence reporting and assessment (mERA) guidelines, including mHealth essential criteria			
**Criteria**	**Item no**	**Notes**	**Page no. where item is reported**
Infrastructure (population level)	1	Clearly presents the availability of infrastructure to support technology operations in the study location. This refers to physical infrastructure such as electricity, access to power, connectivity etc. in the local context. Reporting X% network coverage rate in the country is insufficient if the study is not being conducted at the country level	5,13,23
Technology platform	2	Describes and provides justification for the technology architecture. This includes a description of software and hardware and details of any modifications made to publicly available software	11-12
Interoperability/ Health information systems (HIS) context	3	Describes how mHealth intervention can integrate into existing health information systems. Refers to whether the potential of technical and structural integration into existing HIS or programme has been described irrespective of whether such integration has been achieved by the existing system	24
Intervention delivery	4	The delivery of the mHealth intervention is clearly described. This should include frequency of mobile communication, mode of delivery of intervention (that is, SMS, face to face, interactive voice response), timing and duration over which delivery occurred	13
Intervention content	5	Details of the content of the intervention are described. Source and any modifications of the intervention content is described	8-12
Usability/content testing	6	Describe formative research and/or content and/or usability testing with target group(s) clearly identified, as appropriate User feedback 7	5-8<br>13<br>16-18
User feedback	7	Describes user feedback about the intervention or user satisfaction with the intervention. User feedback could include user opinions about content or user interface, their perceptions about usability, access, connectivity, etc	17,18
Access of individual participants	8	Mentions barriers or facilitators to the adoption of the intervention among study participants. Relates to individual-level structural, economic and social barriers or facilitators to access such as affordability, and other factors that may limit a user's ability to adopt the intervention	12,13,23

Cost assessment	9	Presents basic costs assessment of the mHealth intervention from varying perspectives. This criterion broadly refers to the reporting of some cost considerations for the mHealth intervention in lieu of a full economic analysis. If a formal economic evaluation has been undertaken, it should be mentioned with appropriate references. Separate reporting criterion are available to guide economic reporting	12, 13
Adoption inputs/ programme entry	10	Describes how people are informed about the programme including training, if relevant. Includes description of promotional activities and/or training required to implement the mHealth solution among the user population of interest	13
Limitations for delivery at scale	11	Clearly presents mHealth solution limitations for delivery at scale	23-24
Contextual adaptability	12	Describes the adaptation, or not, of the solution to a different language, different population or context. Any tailoring or modification of the intervention that resulted from pilot testing/usability assessment is described	10,12,15
Replicability	13	Detailed intervention to support replicability. Clearly presents the source code/screenshots/ flowcharts of the algorithms or examples of messages to support replicability of the mHealth solution in another setting	8,9,15,,21
Data security	14	Describes the data security procedures/ confidentiality protocols	12
Compliance with national guidelines or regulatory statutes	15	Mechanism used to assure that content or other guidance/information provided by the intervention is in alignment with existing national/regulatory guidelines and is described	10
Fidelity of the intervention	16	Was the intervention delivered as planned? Describe the strategies employed to assess the fidelity of the intervention. This may include assessment of participant engagement, use of backend data to track message delivery and other technological challenges in the delivery of the intervention	12,17,20,22
